# Three-Dimensional Analysis of Choroidal Vessels in the Eyes of Patients With Unilateral BRVO

**DOI:** 10.3389/fmed.2022.854184

**Published:** 2022-04-05

**Authors:** Lulu Chen, Mingzhen Yuan, Lu Sun, Youxin Chen

**Affiliations:** ^1^Department of Ophthalmology, Peking Union Medical College Hospital, Chinese Academy of Medical Sciences, Beijing, China; ^2^Key Laboratory of Ocular Fundus Diseases, Peking Union Medical College, Chinese Academy of Medical Sciences, Beijing, China; ^3^Beijing Ophthalmology and Visual Sciences Key Laboratory, Beijing Tongren Eye Center, Beijing Tongren Hospital, Capital Medical University, Beijing, China

**Keywords:** branch retinal vein occlusion, choroidal vascularity index, choriocapillaris density, swept source optical coherence tomography, choroidal dilatation

## Abstract

**Purpose:**

To investigate the three-dimensional analysis of choroidal vascular changes in eyes with monocular branch retinal vein occlusion (BRVO) using swept-source optical coherence tomography (SS-OCT).

**Methods:**

Twenty two unilateral BRVO patients with superior-temporal branch retinal vein occlusion and 27 healthy eyes were analyzed retrospectively. OCT and OCT angiography (OCTA) images of 12 * 12 mm centered on the foveal of each eye were analyzed. Three-dimensional choroidal vascularity index (CVI), choroidal thickness, and choriocapillaris density were compared among BRVO eyes, fellow eyes, and healthy control eyes. En face CVI maps in BRVO eyes were generated to analyze the dilatation pattern of choroidal vessels.

**Results:**

CVI values in a few 1 * 1 mm grids in the non-affected hemi side were higher in BRVO eyes compared with the fellow eyes and control eyes (*p* < 0.05). Choriocapillaris density decreased in both BRVO eyes and fellow eyes compared with normal eyes while choriocapillaris density was higher in a few grids in the non-affected hemi side in BRVO eyes compared with fellow eyes (*p* < 0.05). Choroidal dilatation pattern was categorized into four types and inferior choroidal dilatation and posterior pole choroidal dilatation were the major types.

**Conclusion:**

Three-dimensional CVI tended to increase in non-affected hemi side and choroidal vessels tended to dilate in adjacent areas in BRVO eyes. Choriocapillaris density decreased in both eyes of monocular BRVO patients. The choroidal changes suggested that choroidal redistribution occurred in BRVO.

## Introduction

Branch retinal vein occlusion (BRVO) is one of the leading causes of vision loss. It is characterized by retinal hemorrhage, macular edema, neovascularization, and vitreous haemorrhage (1). Previous researches focusing on retinal microvascular changes with optical coherence tomography angiography (OCTA) have reported radial peripapillary capillary drop out (2), decreasing of deep and superficial retinal vascular layer (3, 4), deformation of the foveal avascular zone (FAZ) (5, 6), and formation of microaneurysm and telangiectasia (7). As the swept-source OCTA (SS-OCTA) became available, the choroidal layer was better visualized and studies have been done trying to explore the relationship between changes in choroidal structure and retinal vein occlusion.

Increased subfoveal choroidal thickness in retinal vein occlusion has been reported that tends to decrease after anti-VEGF treatment (8–11). Several studies reported decreased choriocapillaris flow density in retinal vein occlusion eyes (12, 13) and fellow eyes (14). More recently, a few studies have reported a lower choroidal vascularity index (CVI) in retinal vein occlusion eyes (12, 15). Choroidal vascularity index, which was a parameter of quantitative analysis of choroidal structure, has been introduced to evaluate the ratio of the luminal area (LA) to the stromal area (SA) in the binarized image (16, 17). However, the choroidal metrics were only evaluated at the foveal region or on a single B-scan image in previous studies. A thorough three-dimensional evaluation of choroid structure is necessary for understanding the relationship between choroidal alterations and BRVO.

In this study, we investigated the three-dimensional choroidal changes in BRVO eyes with comparison to contralateral fellow eyes and healthy eyes.

## Materials and Methods

### Subjects

This retrospective study included patients diagnosed with branch retinal vein occlusion with a mean duration of 12.3 ± 7.1 months at Peking Union Medical College Hospital from Jan 1st, 2019 to Dec 1th 2021. 22 unilateral BRVO patients with superior-temporal branch retinal vein occlusion were enrolled.

All patients underwent a comprehensive examination including best-corrective visual acuity (BCVA), intraocular pressure, anterior segment, and fundus examination, and swept-source OCT and OCTA (VG200; SVision Imaging, Ltd., Luoyang, China) after complete resolution of macular edema (i.e., after complete resolution of subretinal and intraretinal fluid, and establishment of normal macular contour with intravitreal anti-vascular endothelial growth factor). BRVO patients were excluded if they had diabetic retinopathy, pathologic myopia, glaucoma, or previous surgery excluding phacoemulsification. Patients who have been treated with laser photocoagulation were also excluded. The fellow unaffected eyes of the BRVO patients were also enrolled and have undergone comprehensive examination. The control group consisted of 27 age-matched individuals who have been examined thoroughly and have no history of any ocular diseases or surgery. One eye was randomly selected from each normal individual to consist of the control group.

### Swept-Source Optical Coherence Tomography/OCT Angiography Image Acquisition and Analysis

**FIGURE 1:**
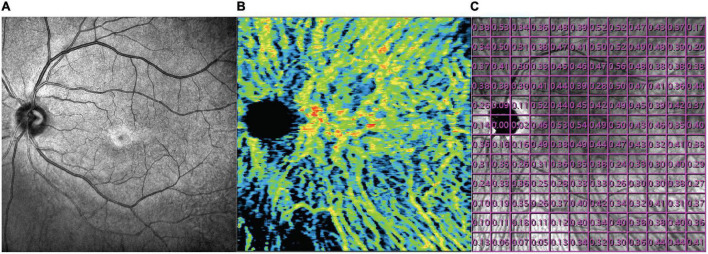
Example of a SLO image **(A)**, choroidal vascularity index image (middle), and the detailed CVI values of each 1 * 1 mm grids **(B)** from the fellow eye **(C)** of a enrolled BRVO patient.

OCT and OCTA images were obtained after total resolution of macular edema using a commercial SS-OCT device (VG200; SVision Imaging, Ltd., Luoyang, China) which contained a SS laser with a central wavelength of approximately 1,050 nm (990–1,100 nm full width) and a scan rate of 200,000 A-scans per second. The OCT and OCTA image of a 12 * 12 mm square centered on the fovea was scanned with the raster scan protocol of 1,024 B-scans, where each B-scan was composed of 1,024 A-scans for each studied eye (resolution of image 1,024 * 1,024). The average thickness of macular fovea was calculated in the inner 1 mm circle of the Early Treatment of Diabetic Retinopathy Study (ETDRS) chart. The choroid was defined as the volume from the basal border of the retinal pigment epithelium-Bruch membrane complex to the choroidoscleral interface. To show the thickness of the choroid layer in detail, average choroidal thickness was calculated in each 1 * 1 mm grid and a 12 * 12 mm thickness map was generated for each eye. The choroidal vascularity index refers to the ratio of the volume of the large and medium choroidal vessels to the volume of the choroid. The VG200D’s van Gogh software uses artificial intelligence algorithms to identify the contours of the large and medium choroidal vessels in the B-scans and then forms the morphology of vessels through three-dimensional reconstruction to realize quantification of the large and medium choroidal vessels. It is a three-dimensional index that reflects the volumetric choroidal vascularity density. A color-coded map was generated to demonstrate the choroidal vascularity index from the en face image and a 12 * 12 mm CVI map was also generated consisting of 144 grids of 1 * 1 mm (Figure 1). The choriocapillaris density refers to the ratio of blood flow to the scanning area at the choriocapillaris layer from the en face image. The choriocapillaris layer was defined as the area between 10 μm above the Bruch’s membrane and 25 μm below the Bruch’s membrane. A choriocapillaris density map of 12 * 12 mm consisting of 144 1 * 1 mm grids was also generated for each studied eye. Since the study area is mirror symmetry for left eyes and right eyes, data for the right eyes was converted for analysis.

### Statistical Analysis

Statistical analysis was performed using the software SPSS 25.0 (IBM, Chicago, United States). Continuous variables were summarized as mean and standard deviation. The chi-square test was used to analyze the distribution of gender and side of enrolled eyes. Wilcoxon signed-rank test was applied to compare macular and choroid metrics between BRVO eyes and fellow eyes. Mann-Whitney U test was applied to compare data between fellow eyes and normal control eyes, and BRVO eyes and control eyes. A *P*-value of < 0.05 was considered statistically significant.

## Results

### Demographic Characteristics

22 unilateral superior-temporal branch retinal vein occlusion patients (10 male, 12 female) were enrolled in this study. The mean duration time of the BRVO patients was 12.3 ± 7.1 months. 18 patients have received intravitreal anti-VEGF treatment with a mean injection of 2.6 ± 2.2 times. 27 eyes from 27 age-matched normal subjects (15 male, 12 female) consisted of the control group. There was no significant difference between the BRVO patients and control groups in terms of age, gender, and refractive error (*p* > 0.05). The BCVA in the BRVO eyes was worse than the fellow eyes (log MAR 0.25 ± 0.27 vs. log MAR 0.00 ± 0.04, *p* < 0.05) and control eyes (log MAR 0.25 ± 0.27 vs. log MAR 0.00 ± 0.02, *p* < 0.05). Mean foveal retinal thickness was 284 ± 27 μm in the BRVO eyes, thicker than the fellow eyes (271 ± 23 μm, *p* < 0.05) (Table 1).

**TABLE 1:** Patient demographic and characteristic.

	Unilateral BRVO					
	BRVO eyes (*n* = 22)	Fellow eyes (*n* = 22)	*P*	Control eyes (*n* = 27)	*P*	*P*
Age (y)	58.8 ± 9.2	58.8 ± 9.2		54.3 ± 9.8	0.106	
Sex (male: female)	10/12	10/12		15/12	0.677**	
Enrolled eye (right: left)	11/11	11/11		16/11	0.719**	
Spherical equivalent (D)	0.79 ± 1.24	0.65 ± 1.21	1.000*	1.13 ± 0.63	0.001^#^	0.416^$^
BCVA (log MAR)	0.25 ± 0.27	0.00 ± 0.04	< 0.001*	0.00 ± 0.02	< 0.001^#^	0.289^$^
Mean foveal retinal thickness (μm)	284 ± 27	271 ± 23	0.013*	276 ± 22	0.640^#^	0.260^$^

*BRVO; branch retinal vein occlusion, D; diopter. Values were presented as mean ± standard deviation.*

*Wilcoxon signed-rank test between BRVO eyes and fellow eyes.

*^#^Mann-Whitney U-test between BRVO eyes and control eyes.*

*^$^Mann-Whitney U-test between fellow eyes and control eyes.*

**Chi-Square test.

### Choroidal Vascularity Index

**FIGURE 2:**
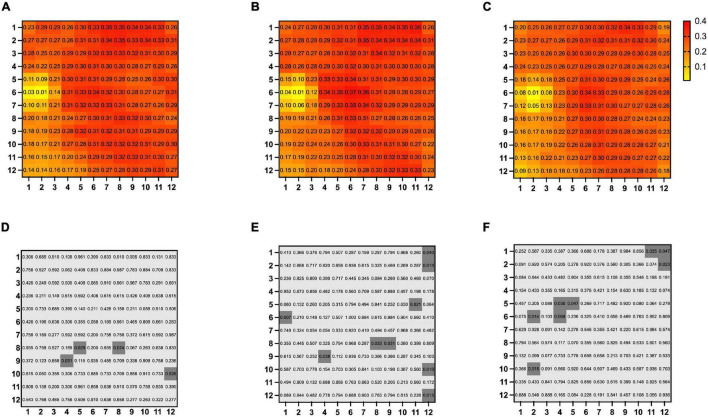
Map of mean choroidal vascularity index 12 * 12 mm grids in BRVO eyes **(A)**, fellow eyes **(B)**, and control eyes **(C)**. Comparison of CVI in each of the 12 * 12 mm grids between BRVO eyes and fellow eyes **(D)**, BRVO eyes and control eyes **(E)** and fellow eyes and control eyes **(F)**. A *p*-value of less than 0.05 was highlighted with a darker gray background. Presented in left eye mode.

**FIGURE 3:**
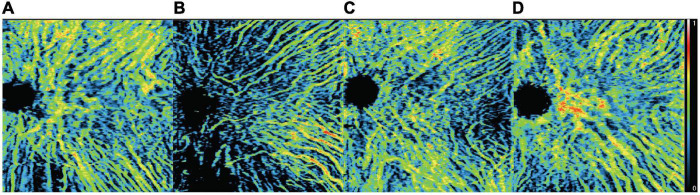
Four patterns of choroidal dilatation. Superior choroidal dilatation **(A)**, inferior choroidal dilatation **(B)**, superior and inferior choroidal dilatation **(C)** and posterior pole choroidal dilatation **(D)**. Presented in left eye mode.

There was no significant difference in mean overall CVI among BRVO eyes, fellow eyes, and control eyes (0.27 ± 0.06 in BRVO eyes, 0.27 ± 0.06 in fellow eyes, and 0.24 ± 0.06 in the control eyes, all *p* > 0.05). Detailed CVI in each 1 * 1 mm grid is presented in Figure 2. CVI values in a few 1 * 1 mm grids were higher in BRVO eyes compared with the fellow eyes (*p* < 0.05) and all those grids were in the lower half section of the scanning area. CVI values in a few grids in the BRVO eyes were higher compared with CVI values in the control eyes (*p* < 0.05). A color-coded map was generated to display the CVI from the en face image for each eye and we categorized the choroidal dilatation into four types according to the location of the dilated choroid vessels in BRVO eyes (Figure 3). Vessel dilatation was defined when a medium or large choroidal vessel shows higher CVI compared with surrounding choroidal vessels. Superior or Inferior choroidal dilatation was defined when choroidal vessels in the corresponding area shows the characteristic of dilation. Posterior pole choroidal dilatation was defined when choroidal vessels had higher CVI in the posterior pole region compared with the surrounding area. Inferior choroidal dilatation was found in 41% of BRVO eyes, superior choroidal dilatation in 9% BRVO eyes, both superior and inferior choroidal dilatation in 14% of BRVO eyes and posterior pole choroidal dilatation in 36% BRVO eyes.

### Choroidal Thickness

The mean overall choroidal thickness in the scanned area was 276.9 ± 60.6 μm in BRVO eyes, 280.8 ± 60.1 in fellow eyes, and 270.9 ± 56.0 in control eyes. There was no significant difference in overall choroidal thickness among BRVO eyes, fellow eyes, and control eyes (*p* > 0.05). Analysis of choroidal thickness in each 1 * 1 mm grid showed no difference among BRVO eyes, fellow eyes, and control eyes (*p* > 0.05).

### Choriocapillaris Density

**FIGURE 4:**
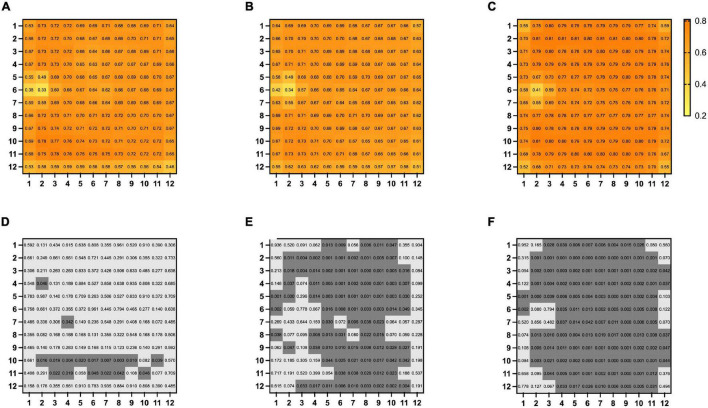
Map of mean choriocapillary density in 12 * 12 mm grids in BRVO eyes **(A)**, fellow eyes **(B)**, and control eyes **(C)**. Comparison of choriocapillaris density in each of the 12 * 12 mm grids BRVO eyes and fellow eyes **(D)**, BRVO eyes and control eyes **(E)** and fellow eyes and control eyes **(F)**. A *p*-value of less than 0.05 was highlighted with a darker gray background. Presented in left eye mode.

The mean overall choriocapillaris density was 0.68 ± 0.06 in BRVO eyes, 0.66 ± 0.07 in fellow eyes, and 0.75 ± 0.11 in control eyes. Overall choriocapillaris densities were lower both in BRVO eyes and fellow eyes compared with control eyes (*p* < 0.05). There was no significant difference in overall choriocapillaris density between BRVO eyes and fellow eyes (*p* > 0.05). In a detailed analysis of choriocapillaris density in 1 * 1 mm grids, we found choriocapillaris density in several grids in the lower half of the scanned area in BRVO eyes was higher than fellow eyes (*p* < 0.05). Choriocapillaris densities in supertemporal and inferotemporal areas were lower in BRVO eyes and fellow eyes compared to normal eyes (*p* < 0.05) (Figure 4).

## Discussion

Branch retinal vein occlusion is a vascular disease caused by occlusive retinal veins which primarily affects retinal blood circulation. Recent studies with SS-OCT or enhanced depth-OCT (EDI-OCT) reported structural changes in the choroidal layer and suggested the involvement of choroid in the pathological alteration of retinal vein occlusion (12, 15, 18). In our study, we measured the structural changes in BRVO eyes with SS-OCT/OCTA in a three-dimension perspective and compared choroidal metrics with the fellow eyes and normal control eyes. We observed that the CVIs were increased in a few grids from the non-occluded hemi side in BRVO eyes compared with fellow eyes and control eyes. Choriocapillaris density decreased significantly in both BRVO eyes and fellow eyes compared with normal eyes. There was no significant difference in terms of choroidal thickness among the three groups. We have also characterized a simplified pattern of choroidal dilatation into four types and found inferior choroidal dilatation was the major type.

Several studies have reported thickened central choroid layer in treatment naïve BRVO eyes which tends to decrease in time with treatment of intravitreal anti-VEGF or steroids (15, 19–21). Du and associates reported that longstanding BRVO eyes without macular edema do not show a significant change of choroidal thickness (9). In our study, we measured choroidal thickness in BRVO patients who have complete resolution of macular edema. Our result shows there was no significant difference in terms of choroidal thickness among BRVO eyes, fellow eyes, and control eyes, which agreed with Du’s work.

Kang’s and Lee’s groups reported similar results of reduction of peripapillary choroidal thickness in both eyes in unilateral BRVO patients (22, 23). Sirayaka and colleagues found a reduction of peripapillary choroidal thickness in superior and inferior quadrants compared to healthy eyes (24). However, peripapillary choroidal thickness did not differ significantly among groups in our study. The different results of our research to others could be due to different timing of measurement. In previous research, peripapillary choroidal thickness was measured at the acute phase of RVO and followed for 6 months or more. The choroidal thickness would increase due to the congestion of retinal circulation and then decreased gradually as the congestion ceases. Besides, previous studies used EDI-OCT images and measured the pinpointing choroidal thickness around the optic nerve head. In our research, we applied the SS-OCT device, which could better visualize the choroid, and we measured the average choroidal thickness of each 1 * 1 mm grid. The different strategies of measurement could be the reason for the difference in our results from others.

Choroidal vascularity index was a newly developed parameter that represents the ratio of the luminal vascular choroid to the total choroid. It has been proved to be a useful indicator of the vascular status of the choroid in various diseases (25–27) and there are a few studies demonstrating a lower CVI in retinal vein occlusion eyes (12, 15, 18). It was largely determined by the medium and large choroid vessels. In these studies, CVIs were calculated in the center subfoveal region with a width of 1,500 μm on a single B-scan of EDI-OCT image. In our study, the three-dimensional CVI was calculated which covered an area of 12 * 12 mm centered on the foveal. A total of 144, 1 * 1 mm grids consisted of the study area of each eye and the CVI of each grid was presented to provide a thorough assessment of CVI in studied eyes. Our study showed a higher CVI in a few grids in the non-affected hemi side of BRVO eyes compared with the fellow eyes, and higher CVI in a few grids in the non-affected hemi side and periphery region in BRVO eyes compared with control eyes. There was no significant difference in CVIs in the foveal region among the three groups. In Alis’s and Hwang’s works, they evaluated BRVO eyes with macular edema and proposed that the decreased CVI could be the result of the fluid shift from the retina to choroidal stroma (15, 18). Aribas and associates enrolled patients with complete resolution of macular edema but they enrolled both CRVO and BRVO patients (12). The different criteria of enrollment could be the reason for the different results about the subfoveal CVI of our study to others. In our research, we enrolled only BRVO patients with superior temporal branch retinal vein occlusion and complete resolution of macular edema. We found higher CVI in a few grids in the lower half of the choroid in BRVO eyes which might suggest the tendency of choroidal vessel dilatation in adjacent areas of the occluded region. This may imply large and medium choroid vessels from the adjacent area of occluded regions assisted in blood drainage from affected areas. Studies that further evaluate the drainage pattern of choroidal vessels in a time-dependent manner are urgent in the future. From the color-coded en face CVI map, choroidal dilatation was better visualized and we categorized the choroidal dilatation pattern into four types. Inferior choroidal dilatation was the most common type (41%) which was followed by posterior pole choroidal dilatation (36%). The drainage route in RVO has not been fully revealed yet and based on our findings it is reasonable to suggest that adjacent choroid vessels participated in draining the stagnant blood in occluded regions. However, our study is a cross-sectional study, and grids with higher CVI were also limited. Studies with close follow-up of the choroid layer in BRVO eyes are needed to better elucidate the drainage pattern of the choroid in BRVO eyes.

Decreased choriocapillaris density in the macular region or peripapillary region has been reported in RVO eyes and fellow eyes in recent years (12, 14, 28, 29). Some researchers suggested that the decrease of choriocapillaris density in the macular region was caused by hyperexpression of VEGF (30) while Aribas and associates believed choriocapillaris drop out in the macular region could be the pressure effect of the choroidal congestion (12). As for the decrease of peripapillary choriocapillaris density, the vascular theory has been proposed by researchers (22, 31) which suggested that the disturbance of peripapillary blood circulation would eventually lead to glaucoma. In a most recent study by Park and associates, the proportion of signal voids in the peripapillary choroid layer was larger in uninvolved fellow eyes of BRVO patients than in control eyes (29). The etiology of decreased peripapillary choroid flow has not been well elucidated, but the author suggested that the decreased choroid flow represented pathogenic circumstances, which made the eye more susceptible to RVO. Overall choriocapillaris densities in BRVO and fellow eyes were lower than normal control eyes in our study. In a detailed analysis, grids with lower choriocapillaris density in BRVO and fellow eyes were partially encompassing the foveal and the peripapillary area. The underlying mechanism of general choroidal capillary decreasing in both eyes of unilateral BRVO is not clear, but we think that the decreased capillary density may represent the result of vascular alteration influenced by a variety of systemic factors. Interestingly, choriocapillaris density in a few grids in the lower half of BRVO eyes is higher than the fellow eyes. We hypothesized that the retinal circulation has reconstructed after BRVO occurred and the choroid circulation has been redistributed along with the changes of retinal circulation. However, the current study only enrolled limited BRVO patients, and studies with a larger BRVO population would be needed to further evaluate changes in choroid vasculature in the future.

To the best of our knowledge, there have been limited studies on choroidal changes related to BRVO. In most studies, the enrolled subjects were a mixed population of BRVO and CRVO patients. Those that contained only BRVO patients did not specify the affected blood vessel. In our study, we only included BRVO patients with superior-temporal branch retinal vein occlusion so we can get a better picture of the localized changes of choroid circulation. Besides, we used a three-dimensional CVI to evaluate the choroidal changes in detail, which has never been applied in studies of BRVO before and the three-dimensional CVI map provides us with a visualized diagram showing the dilatation of choroidal vessels. The present study thoroughly evaluated changes in the choroid layer from three different perspectives and the large study area of 12 * 12 mm provided us with more information and help us to understand the possible role of the choroid in the development of BRVO.

However, there were limitations of the present study. The study was a retrospective study and only a small number of patients were included. Complete resolution of macular edema was necessary for the enrolled patients and some patients never received intravitreal anti-VEGF treatment due to the absence of macular edema, so the severity of BRVO in our research might be relatively milder. Studies with a larger sample and including BRVO with different severity and different occluded vessels are needed to further investigate the vascular changes in BRVO. Besides, a cohort study with longer continuous follow-up would be more helpful to reveal the redistribution of choroid circulation in the disease course of BRVO.

## Conclusion

In conclusion, the present study demonstrated that three-dimensional CVI in non-affected hemi-side tended to increase and choroidal vessels in adjacent areas of the occluded region in BRVO eyes tended to dilate. Choriocapillary density decreased in both eyes of monocular BRVO patients. The choroidal changes suggested that choroidal redistribution occurred in BRVO.

## Data Availability Statement

The raw data supporting the conclusions of this article will be made available by the authors, without undue reservation.

## Ethics Statement

The studies involving human participants were reviewed and approved by Institutional Review Board of PUMCH. The patients/participants provided their written informed consent to participate in this study.

## Author Contributions

LC and MY contributed to the conception of the protocol. LC and LS collected the data. LC, MY, and LS analyzed the data. LC contributed to writing the first draft of the manuscript. MY contributed to revising the manuscript. YC commented on the manuscript and gave the final approval. All authors contributed to the article and approved the submitted version.

## Conflict of Interest

The authors declare that the research was conducted in the absence of any commercial or financial relationships that could be construed as a potential conflict of interest.

## Publisher’s Note

All claims expressed in this article are solely those of the authors and do not necessarily represent those of their affiliated organizations, or those of the publisher, the editors and the reviewers. Any product that may be evaluated in this article, or claim that may be made by its manufacturer, is not guaranteed or endorsed by the publisher.
